# Enhancing radiology workflows through collaborative AI-assisted chest X-ray reporting using large vision-language models: a proof-of-concept study

**DOI:** 10.1186/s13244-026-02292-7

**Published:** 2026-04-28

**Authors:** Chantal Pellegrini, Ege Özsoy, Florian T. Gassert, Alexander W. Marka, Maximilian Strenzke, Matthias Keicher, Marcus R. Makowski, Nassir Navab

**Affiliations:** 1 School of Computation, Information and Technology, Technical University of Munich, Munich, Germany; 2 Munich Center of Machine Learning, Technical University of Munich, Munich, Germany; 3 Institute for Diagnostic and Interventional Radiology, School of Medicine and Health, TUM Klinikum, Technical University of Munich (TUM), Munich, Germany

**Keywords:** Artificial intelligence, Chest X-ray, Radiology reporting, Human-AI-collaboration, Large language models

## Abstract

**Objectives:**

To evaluate whether collaborative assistance from an artificial intelligence-based tool that proposes partial radiology report content can improve reporting efficiency and radiologist satisfaction in chest X-ray interpretation, without compromising report quality.

**Materials and methods:**

In a retrospective study, three radiologists reported 50 MIMIC-CXR chest X-rays twice, once with artificial intelligence (AI) assistance and once without. A specialized large vision-language model (LVLM) provided real-time suggestions, which could be accepted, modified or rejected. The study evaluated writing time, suggestion acceptance, report length and quality and assessed usability and suggestion quality on a 5-point Likert-scale questionnaire. Statistical analysis used paired *t*-tests or Wilcoxon signed-rank tests based on normality.

**Results:**

AI assistance reduced mean writing time by 7.80% (*p* = 0.08), with significant gains for complex reports (18.34%, *p* < 0.001). Efficiency improvements correlated with suggestion acceptance and were user-dependent, with benefits up to 27.24% (CI: [17.34, 37.14], *p* < 0.001) for radiologists with high acceptance. Report quality and length remained stable, indicating preserved diagnostic accuracy without degradation. Radiologists rated the tool highly for ease of use (mean: 4.33) and desired regular use (mean: 4), noting minimal errors (mean: 1.67).

**Conclusion:**

Collaborative AI assistance with an LVLM can improve reporting efficiency if well adopted, particularly for complex cases, without compromising quality, and is well-received by radiologists. These exploratory findings suggest potential to optimize radiology workflows through collaborative reporting and warrant prospective validation in clinical settings.

**Critical relevance statement:**

This study critically evaluates a collaborative AI-assisted reporting tool for chest X-rays, demonstrating its potential to enhance radiologist efficiency without compromising automatically measured report quality, thereby demonstrating a potential path for practical integration of AI into clinical radiology workflows.

**Key Points:**

- A collaborative vision-language model supported radiology workflow is proposed, and its effectiveness is studied in a user study.
- Mean writing time for a radiology report decreases with AI support without affecting report quality.
- The AI-assisted tool was rated highly for usability and integration into clinical workflow, supporting its practical adoption in radiology reporting.

**Graphical Abstract:**

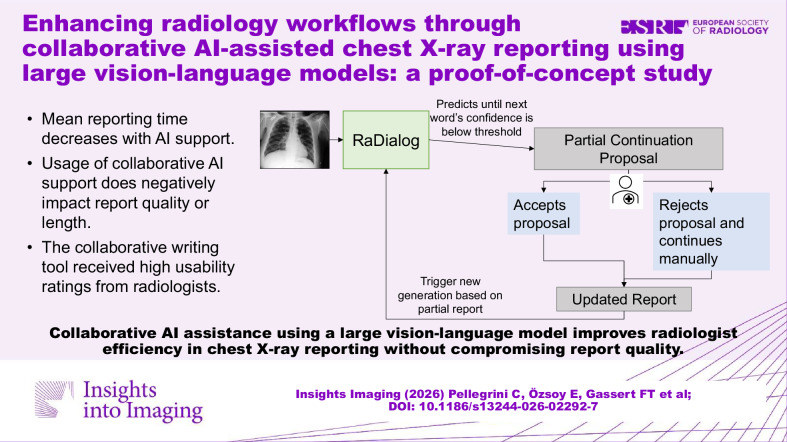

## Introduction

Radiology plays a central role in clinical decision-making, with radiology reports as the primary communication channel between radiologists and other clinicians, such as for chest X-rays, which are critical for diagnosing a variety of pulmonary diseases [1]. However, increasing imaging volumes and a persistent radiologist shortage strain existing resources and demand more efficient reporting solutions [2, 3].

Automated AI-based report generation presents a promising approach to enhance reporting speed and support accurate diagnostic decision-making [2]. However, fully automated end-to-end models are not yet sufficiently reliable, occasionally producing factual inaccuracies or omissions [4]. Thus, they require careful radiologist review before clinical use.

The emergence of large language models (LLMs) has marked a major step in AI, enabling sophisticated reasoning, fluent interactions, and specialized knowledge processing across domains [5]. Within healthcare, these models have effectively addressed challenging applications, including clinical knowledge retrieval, solving medical exams, and interactive clinical decision support [5–10]. Large Vision-Language models (LVLMs), which jointly interpret textual and visual data, have further enhanced the potential of automated radiology report generation [11–13]. However, current approaches predominantly focus on full report generation or basic chat functions [11, 12], without exploring collaborative reporting, allowing incomplete or imperfect proposals by the AI tool, and letting the radiologist interactively accept, edit, or ignore proposals in real time. Unlike one-pass AI pre-reads, this paradigm maintains continuous human oversight and iterative control over the final report.

Some prior studies evaluated AI assistance in radiology workflows, showing promising improvements in both diagnostic performance and efficiency, with AI-aided chest X-ray diagnosis increasing radiologists’ diagnostic correctness [14–16] and reducing mean reading time [14, 15]. While these studies confirm the potential of AI in supporting diagnostic accuracy and reporting speed, they assume static workflows where AI suggestions are presented all at once before human input.

To address this gap, we propose a more dynamic and collaborative framework for AI-assisted reporting. Instead of relying on one-time AI proposals followed by posthoc human correction, our study introduces a collaborative paradigm in which an LVLM provides real-time, context-sensitive suggestions during report writing. By integrating interactive feedback and iterative refinement, such a system could enhance accuracy, align better with radiologists’ preferences, and enable real-time support while maintaining human oversight and flexibility.

In this study, we propose a framework that provides real-time suggestions during report writing by an LVLM, which the radiologist can flexibly decide to use or ignore. We hypothesize that this approach can reduce reporting time and mental effort while improving radiologist satisfaction and workflow efficiency. To evaluate its effectiveness, we conduct a controlled user study with practicing radiologists, assessing its impact on reporting speed, quality, and usability.

## Methods

### Study design and data

This retrospective study evaluates the effectiveness of AI-assisted report generation in a collaborative setting. We use the MIMIC-CXR dataset [1], a publicly available, de-identified collection of 377,110 chest X-rays and associated reports. From the test set, we randomly selected 50 scans with a non-empty Findings section to ensure baseline availability for text-based evaluation; this criterion targets text presence rather than abnormality and does not exclude normal cases. Only test data were used to avoid model exposure during training.

### Reader study

Three radiologists participated in the study: Radiologist 1 (board-certified, 8 years of experience), Radiologist 2 and 3 (residents, 4 and 3 years). Each radiologist independently interpreted all 50 X-ray images twice, once without assistance and once with AI assistance, with a randomized order of scans to minimize recall bias. Although no formal washout period was used, reading sessions were distributed across multiple days, and case order was randomized and counterbalanced so that AI-assisted and unassisted reads were equally likely to occur first or second, mitigating systematic recall bias. During unassisted reads, reports were composed free-text in the same interface with all AI features disabled; no structured templates or auto-completions were available. Reading sessions were conducted on clinical-grade PACS workstations using DICOM images. Radiologists were blinded to each other’s reports and, during unassisted reads, to AI outputs. They received brief instructions on tool usage before the study.

### Report generation model

The RaDialog model [13], which is publicly available on HuggingFace [17], was used for generating AI proposals. RaDialog is an LVLM for chest X-ray report generation, featuring a dual-branch architecture: a Visual Feature Extractor that converts the X-ray into image tokens in the LLM token space and a Structured Findings Extractor that predicts key radiological findings (e.g., lung opacity, edema, cardiomegaly), which are converted into a short text description. During inference, RaDialog considers a multimodal input from (1) the image tokens, (2) the structured-findings text, and (3) the current partial report. The LLM then generates subsequent tokens conditioned on this joint context, so that every suggested word is based on the X-ray–derived image tokens and structured findings as well as the partial report. The model employs a fine-tuned LLM, trained on ~580k instruction-style samples for tasks including report generation, question answering, and correction. The model and its dataset are publicly available and rank high in report generation leaderboards, providing a good foundation for our collaborative writing approach. We prompt the model as described in the RaDialog paper to generate a report for the given image and additionally include all previously generated parts of the current report in the prompt at each proposal step.

### Collaborative AI assistance

**Fig. 1:**
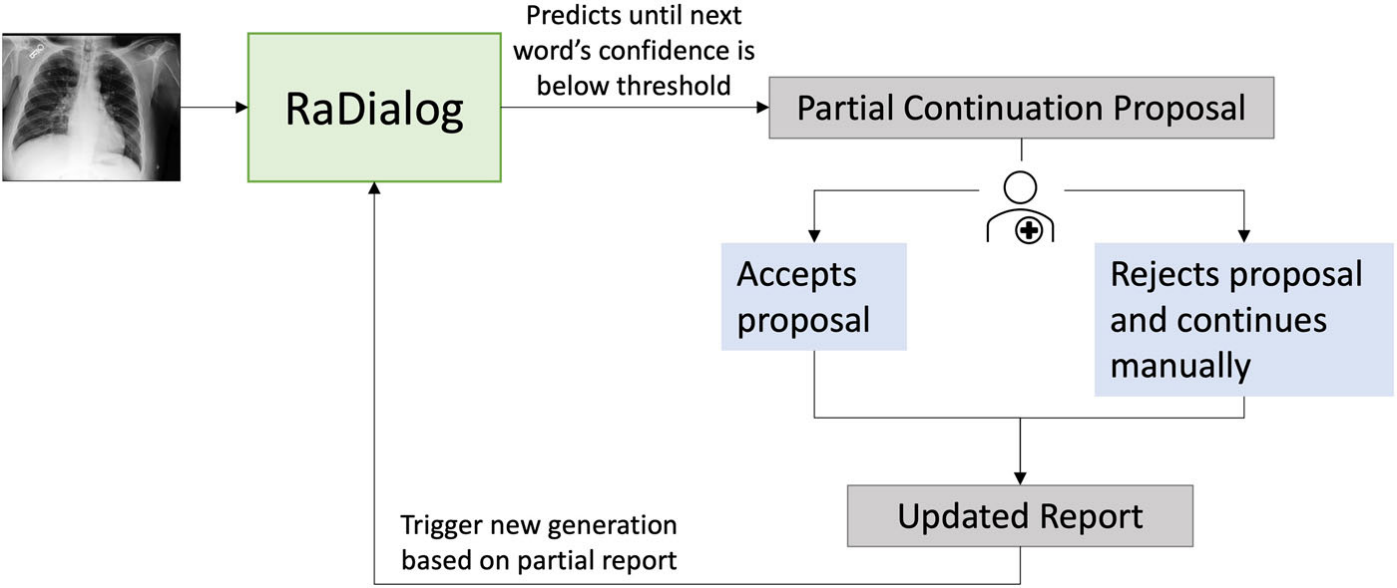
Overview of the proposed collaborative reporting system. The system continuously predicts the next word in the report until the model’s confidence for the upcoming token falls below a predefined threshold. At that point, RaDialog presents a partial continuation proposal to the user. If the proposal is accepted, the system incorporates the suggested text; if rejected, the user manually continues the report. Upon acceptance or manual revision, the report is updated and triggers a new generation cycle based on the current report state, thereby iteratively refining the text while maintaining user oversight

**Fig. 2:**
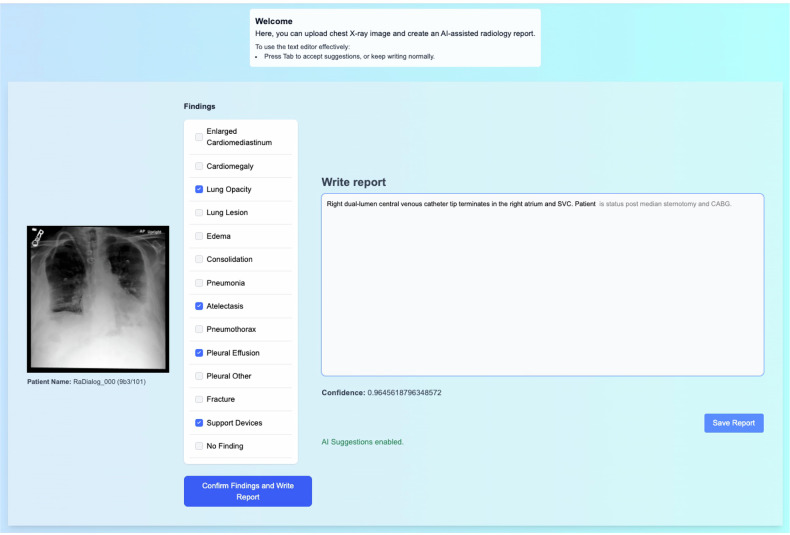
Screenshot of the developed collaborative reporting tool used in the study. The left pane shows the current image, which the radiologist additionally sees in their DICOM viewer. Given the image, the users can first adjust and accept the high-level findings provided by RaDialog in the middle panel. Afterward, they start typing a report in the “Write report” field. The suggested partial report continuations are shown in semi-transparent gray text, and the user can choose to accept them by pressing “Tab” or reject the AI-generated segment by continuing to type. If accepted, the proposed text is inserted into the report. This interface design ensures seamless collaborative report writing, integrating AI suggestions with user input, fostering efficient yet controlled report generation

We built an interactive tool using RaDialog [13, 17] to assist radiologists in drafting chest X-ray reports efficiently. Figure 1 illustrates our collaborative workflow. Designed as a locally hosted web-based platform (Fig. 2), the interface displays the X-ray image on the left, linked to its DICOM ID for viewing on a calibrated monitor, and a text field on the right for report drafting. The tool leverages RaDialog’s dual-branch architecture to initially suggest high-level findings, which radiologists can review and adjust. As typing begins, our tool provides real-time predictive suggestions that appear unobtrusively in light gray, which can be accepted with Tab or discarded by continuing to type. Suggestions are generated within 1 s, ensuring low-latency interactions. Model suggestions are displayed only when the model’s confidence, derived from the SoftMax probability of the predicted tokens, exceeds a threshold of 0.5, ensuring more reliable completions. This confidence is recalculated after each word, limiting suggestions to high-probability sequences.

To support analysis, we logged suggestion counts, acceptances, modifications, writing time (from first to last keystroke), and report length using JavaScript.

### Questionnaire design

**Fig. 3:**
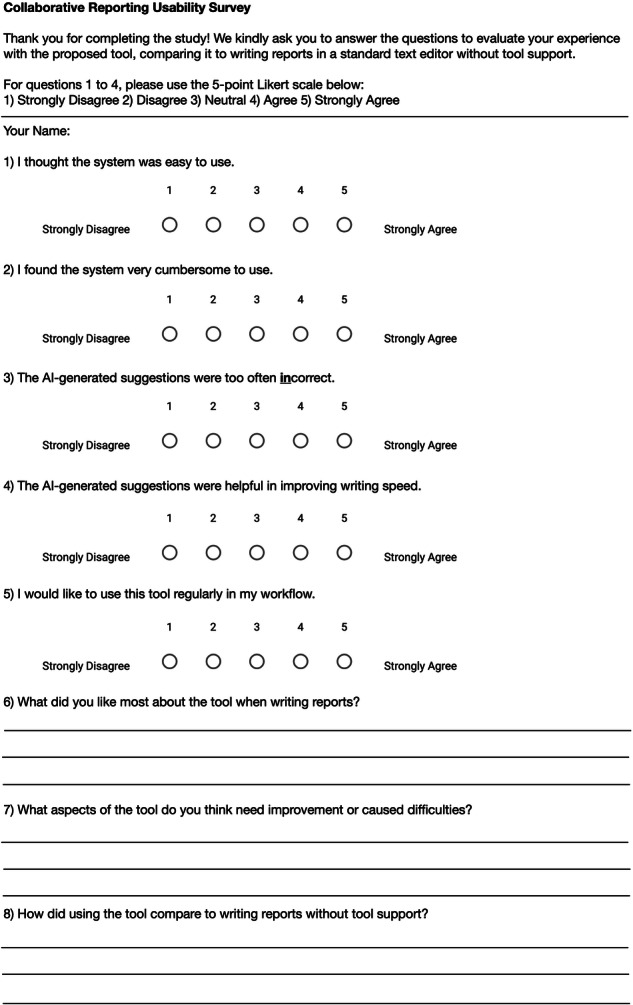
Usability questionnaire evaluating the proposed collaborative reporting system. Printed questionnaire given to radiologists after completing all study samples. Sections 1–5 employ a 5-point Likert scale (1 = strongly disagree to 5 = strongly agree) to assess system ease of use, perceived cumbersomeness, frequency of incorrect AI suggestions, effect on writing speed, and willingness to adopt the tool in routine workflow. Sections 6–8 comprise open-ended questions soliciting qualitative feedback on the tool’s most appreciated features, suggested improvements or encountered difficulties, and comparative experiences with conventional report writing without AI support. This instrument quantitatively and qualitatively evaluates user satisfaction and identifies usability issues to guide future work

To evaluate radiologists’ subjective experience with AI assistance, we designed a usability survey. The questionnaire, shown in Fig. 3, was developed based on usability frameworks from human-computer interaction literature [18] and tailored to radiology reporting needs. All questions were rated on a 5-point Likert scale (1 = strongly disagree, 5 = strongly agree) assessing ease of use, suggestion accuracy, and workflow integration, alongside three open-ended questions for qualitative feedback on most appreciated features, difficulties and improvement potential, and comparison to non-AI-assisted workflows.

### Evaluation and statistical analysis

Continuous variables were summarized using means with 95% confidence intervals (CIs). Statistical significance was defined as *p* < 0.05. Normality was assessed via the Shapiro–Wilk test. Paired *t*-tests were used for normally distributed data (e.g., writing time); Wilcoxon signed-rank tests were used otherwise (e.g., BLEU-1).

In evaluating report quality, we employ standard natural language generation metrics, including BLEU-1 [19], METEOR [20], and ROUGE-L [21], alongside the macro-averaged CheXbert F1 score for assessing diagnostic accuracy as described by Smit et al [22], comparing participants’ reports against MIMIC-CXR ground-truth reports. This approach is intended to assess whether AI support influences report quality, rather than to serve as an absolute quality benchmark, given the inherent variability in X-ray reporting. Character count was used to assess verbosity.

For the bucket analysis, reports were stratified into two discrete groups based on unassisted writing time to approximate baseline complexity. Within each bucket, mean writing times were compared using confidence intervals and paired significance tests.

For suggestion metrics, we recorded the total number of AI-generated suggestions, their acceptance and rejection count and percentage.

Radiologist feedback was reported per individual. For negatively phrased items, lower scores indicated more favorable responses.

The stats module of the scipy library, version 1.11.3, and custom Python 3.10.13 scripts were used to perform all statistical analyses. No formal power calculation was performed, as this study was exploratory in nature. The sample size was limited by feasibility and intended for initial insight.

## Results

The evaluation compared radiologists’ performance under assisted and unassisted conditions across suggestion interaction behavior, time efficiency, report quality, and subjective user satisfaction. Below, we present all quantitative results and qualitative radiologist feedback.

### Suggestions and AI text contribution

AI suggestion metrics are detailed in Table 1. Radiologist 1 accepted 6.38 suggestions per report (CI: [5.55, 7.21]), rejected 3.32 (CI: [2.73, 3.91]), with an acceptance rate of 68.43% (CI: [63.79, 73.08]). Radiologist 2 accepted 7.28 (CI: [6.10, 8.45]), rejected 4.28 (CI: [3.32, 5.24]), with a 65.82% rate (CI: [59.60, 72.04]). Radiologist 3 accepted fewer at 2.70 (CI: [2.17, 3.23]), rejected 5.25 (CI: [3.85, 6.65]), with a 40.72% rate (CI: [32.22, 49.23]). Overall, 5.61 suggestions were accepted (CI: [5.01, 6.22]), 4.21 rejected (CI: [3.64, 4.78]), yielding a 59.45% acceptance rate (CI: [55.31, 63.58]).

**Table 1:** Suggestion utilization metrics [95% CI]

Radiologist	Accepted	Rejected	Accept. rate (%)
R1	6.38 [5.55, 7.21]	3.32 [2.73, 3.91]	68.43 [63.79, 73.08]
R2	7.28 [6.10, 8.45]	4.28 [3.32, 5.24]	65.82 [59.60, 72.04]
R3	2.70 [2.17, 3.23]	5.25 [3.85, 6.65]	40.72 [32.22, 49.23]
Overall	5.61 [5.01, 6.22]	4.21 [3.64, 4.78]	59.45 [55.31, 63.58]

### Efficiency metrics

**Fig. 4:**
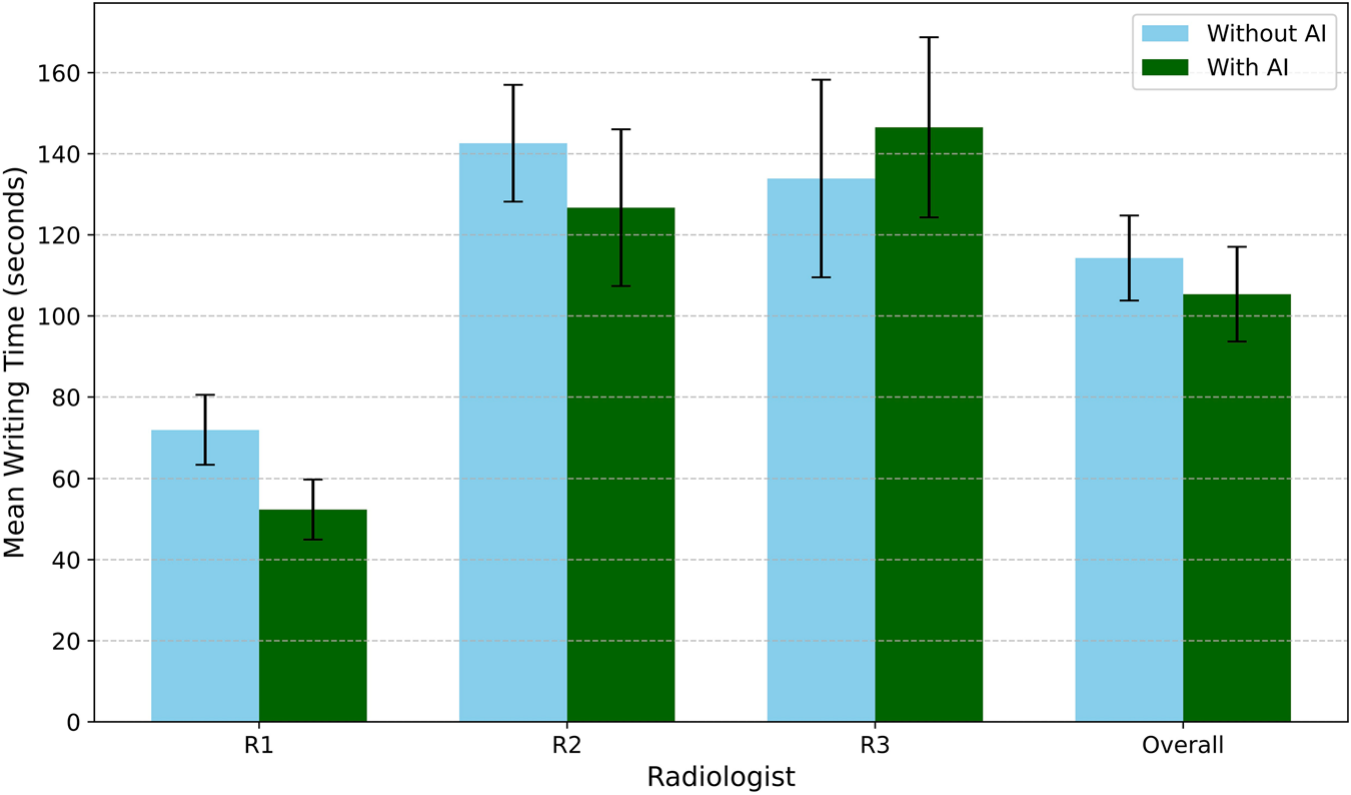
Mean report writing times with and without AI assistance. Bar-chart comparison of mean report writing times in seconds for three radiologists (R1–R3) and the overall average under two conditions: manual reporting without AI assistance (blue bars) versus AI-assisted reporting with RaDialog (green bars). AI support yielded time savings for two of three users, reducing the mean writing times overall and demonstrating the efficiency benefits of integrated model-based suggestions during report composition. Error bars are 95% CI

We assessed efficiency by comparing writing times between AI-assisted and non-AI conditions across three radiologists (Table 2, Fig. 4).

**Table 2:** Writing time efficiency metrics

Radiologist	AI (s)	No AI (s)	Diff. (%) [*p*]
R1	52.37 [44.96, 59.77]	71.97 [63.41, 80.54]	27.24 [17.34, 37.14] [< 0.001]
R2	126.71 [107.38, 146.03]	142.60 [128.19, 157.00]	11.14 [−0.69, 22.98] [0.070]
R3	146.52 [124.30, 168.74]	133.89 [109.52, 158.26]	−9.43 [−29.60, 10.73] [0.323]
Overall	105.36 [93.68, 117.05]	114.28 [103.79, 124.77]	7.80 [−0.72, 16.32] [0.080]

For Radiologist 1, mean writing time decreased significantly from 71.97 s (CI: [63.41, 80.54]) without AI to 52.37 s (CI: [44.96, 59.77]) with AI, a 27.24% reduction (CI: [17.34, 37.14], *p* < 0.001, paired *t*-test). Radiologist 2 showed a reduction from 142.60 s (CI: [128.19, 157.00]) to 126.71 s (CI: [107.38, 146.03]), a difference of 11.14% (CI: [−0.69, 22.98], *p* = 0.07). Radiologist 3 experienced an increase from 133.89 s (CI: [109.52, 158.26]) to 146.52 s (CI: [124.30, 168.74]), a difference of −9.43% (CI: [−29.60, 10.73], *p* = 0.32). Overall, mean writing time was 114.28 s (CI: [103.79, 124.77]) without AI versus 105.36 s (CI: [93.68, 117.05]) with AI, a 7.80% reduction (CI: [−0.72, 16.32], *p* = 0.08).

### Bucket analysis

The bucket analysis categorizes reports into two distinct groups based on unassisted writing time with user-specific thresholds, enabling structured comparison of AI’s impact across different report complexities or lengths. AI assistance had different effects on writing time depending on the report category. In Bucket one, AI use slightly increased writing time by 12.74% on average, though the difference was not statistically significant (*p* = 0.16). In contrast, Bucket two showed a significant reduction in writing time with AI, saving 18.34% on average (*p* < 0.001). In Appendix C, we further report an exploratory finding-based stratification (normal vs abnormal, single- vs multi-finding, and selected finding groups). The largest time reduction is observed for single-finding studies (≈22.8% mean reduction; 95% CI 11.7–33.7), while other groups show smaller effects.

### Report generation quality

AI-assisted reports showed similar performance in NLP-based quality metrics compared to non-AI reports (Table 3). Overall BLEU-1 scores were 0.27 (CI: [0.25, 0.29]) with AI versus 0.25 (CI: [0.23, 0.26]) without AI, METEOR scores were 0.13 (CI: [0.12, 0.13]) versus 0.12 (CI: [0.11, 0.12]), and ROUGE-L scores were 0.24 (CI: [0.23, 0.25]) versus 0.22 (CI: [0.21, 0.23]) (Table 3). CheXbert F1 scores were 0.37 (CI: [0.31, 0.42]) with AI versus 0.40 (CI: [0.32, 0.46]) without AI. Report length was also stable, with an average of 275.33 characters (CI: [259.22, 291.44]) without AI and 278.48 characters (CI: [262.45, 294.51]) with AI (difference: −3.15, CI: [−16.31, 10.00], *p* = 0.64). Further, in Appendix A, we include an ablation analyzing how much the report generation proposals depend on the X-ray image versus the report prefix alone. The findings demonstrate that the suggestions used in the collaborative tool are clearly conditioned on image information, though they are not exclusively constrained by it and can be influenced by learned language priors.

**Table 3:** Report generation quality metrics: Bleu, Meteor and Rouge are NLP-based metrics, while CheXbert F1 is a classification-based metrics comparing major findings in the reports

With AI [95% CI]				
Radiologist	BLEU-1	METEOR	ROUGE-L	CheXbert F1
R1	0.22 [0.19, 0.25]	0.11 [0.10, 0.12]	0.22 [0.20, 0.24]	0.32 [0.24, 0.38]
R2	0.35 [0.31, 0.38]	0.15 [0.14, 0.17]	0.28 [0.26, 0.30]	0.42 [0.29, 0.50]
R3	0.24 [0.21, 0.28]	0.12 [0.10, 0.13]	0.22 [0.20, 0.24]	0.35 [0.26, 0.42]
Overall	0.27 [0.25, 0.29]	0.13 [0.12, 0.13]	0.24 [0.23, 0.25]	0.37 [0.31, 0.42]

Without AI [95% CI]				
Radiologist	BLEU-1	METEOR	ROUGE-L	CheXbert F1
R1	0.22 [0.19, 0.24]	0.11 [0.10, 0.11]	0.21 [0.19, 0.22]	0.33 [0.25, 0.40]
R2	0.32 [0.30, 0.35]	0.14 [0.13, 0.15]	0.25 [0.23, 0.27]	0.38 [0.26, 0.44]
R3	0.20 [0.17, 0.23]	0.10 [0.09, 0.11]	0.21 [0.19, 0.22]	0.45 [0.28, 0.52]
Overall	0.25 [0.23, 0.26]	0.12 [0.11, 0.12]	0.22 [0.21, 0.23]	0.40 [0.32, 0.46]

Difference (AI − non-AI) [*p*]				
Overall	0.02 [0.024]	0.01 [0.004]	0.02 [0.003]	−0.03 [0.420]

### Radiologist feedback

Feedback was collected via a usability questionnaire addressing ease of use, suggestion accuracy, writing speed, desire for regular use, and system cumbersomeness. As shown in Table 4, ease of use was rated highly, with scores of 5, 4, and 4. Writing speed improvement was rated 4, 5, and 4, while all radiologists indicated a score of 4 for their desire to use the system regularly. AI suggestion errors were rated 1, 2, and 2, while system cumbersomeness received scores of 1, 2, and 1. For these negatively phrased items (i.e., “AI suggestion errors” and “system cumbersomeness”), lower scores indicate more favorable evaluations.

**Table 4:** Radiologist questionnaire scores (scale: 1–5)

Question	R1	R2	R3	Mean
Ease of use	5	4	4	4.3
AI suggestion errors↓	1	2	2	1.7
Writing speed improvement	4	5	4	4.3
Desire for regular use	4	4	4	4.0
System cumbersomeness↓	1	2	1	1.3

↓ For negatively phrased items, lower scores indicate more favorable evaluations

## Discussion

This study evaluated the potential of collaborative AI-assisted chest X-ray reporting, focusing on efficiency, report quality, and user experience. Building upon a SOTA report generation system [13], we proposed an interactive tool for partial report completion based on model confidence. Our primary findings demonstrate that AI assistance reduces writing time by an average of 7.80% (from 114.28 s to 105.36 s, *p* = 0.08) without significantly impacting report quality or length. Radiologist feedback further supports the system’s value, rating the tool highly for usability and satisfaction, supporting its clinical feasibility.

Efficiency gains varied among radiologists: two saved writing time, while for one, writing time on average increased. This variability may relate to individual differences in reporting style and the extent to which radiologists accept AI-generated suggestions. Differences in acceptance rates (68.43%, 65.82%, 40.72%) suggest that higher engagement with AI proposals leads to greater efficiency. While efficiency gains remain radiologist-dependent, even partial adoption can offer value through selective, opt-in use by radiologists who demonstrably benefit from the tool.

Bucket analysis showed additional variability by case complexity, with significant reductions for longer, more complex reports (Bucket 2), and a slight, non-significant increase (12.74%, *p* = 0.16) for shorter reports. This indicates that AI benefits may scale with complexity, potentially due to the model’s ability to provide structured suggestions for multifaceted findings.

Using automated report correctness metrics, we observed broadly stable text similarity and near-stable label metrics with a small decline likely driven by phrasing rather than clinical content, given that radiologists finalized all reports. Report verbosity did not change, suggesting that AI suggestions did not inflate documentation. These findings indicate that assistance did not materially alter the textual character of reports; nevertheless, it is important to note that the evaluation relied on automated comparisons to reference reports, not external radiologist reviews.

The ablation in Appendix A indicates that image input guides the model’s proposals but does not fully constrain them, implying that language priors and the written report prefix can contribute to fluent continuations. While this dependence on the report prefix is essential for interactivity, allowing suggestions to stay consistent with the radiologist’s draft, in low-quality or ambiguous studies (e.g., portable ICU radiographs), this same reliance on priors may increase the risk of overly plausible, insufficiently image-grounded text. The proposed workflow mitigates this by keeping the radiologist in full control, but future work could explore performance under image degradation and incorporate safeguards such as quality-aware gating.

Qualitatively, radiologists valued the tool’s integration into their workflow, rating it as intuitive, accurate, and minimally intrusive. In free-text feedback, all participants reported reduced effort for writing and perceived time savings. Other comments mentioned that AI-generated descriptions are complete and follow common formulations. These findings align with the hypothesis that collaborative AI can reduce mental effort and reporting time while maintaining diagnostic reliability. Suggested improvements included enabling full-report drafts when confidence is high and integrating speech-based input to better fit clinical practice.

By combining AI speed with radiologist expertise, the tool addresses increasing workload without sacrificing quality. It functions reliably as a drafting assistant—avoiding the pitfalls of full automation—while aligning with clinical workflows and user preferences. The positive feedback on usability and the expressed desire for regular use underscore the system’s potential to enhance workflow efficiency and radiologist satisfaction, especially for complex cases where time savings are most pronounced.

While these findings show promise, the performed study has limitations. The sample size was restricted to 50 chest X-rays from MIMIC-CXR and three radiologists, limiting generalizability across populations, imaging modalities, or reporting styles. Moreover, our baseline did not include structured templates or phrase libraries, support commonly used in clinical reporting. As such, absolute time savings may differ in template-rich environments, motivating future comparisons against such workflows. Additionally, only one model was tested, and its performance may not reflect that of other LVLMs. Lastly, all participants were rather young, with 3–8 years of experience and likely more familiar with digital tools than senior radiologists. They may be more willing to integrate suggestions directly while drafting, whereas more senior radiologists may have stricter reporting routines and prefer to use AI mainly for final checks. Further, less experienced readers could be more prone to accepting AI suggestions quickly, while very experienced readers may ignore them unless they strongly match their own assessment and preferred wording. Future studies should expand the sample size, include more radiologists to analyze experience-related adoption and usage patterns and compare multiple models to validate these findings.

In summary, this study demonstrates that AI-assisted radiology reporting with RaDialog can enhance efficiency—particularly for complex reports—without degrading quality or length, while earning strong approval from radiologists for its usability and accuracy. Our findings indicate a promising route to optimizing reporting under resource constraints, warranting prospective in-workflow validation.

## Supplementary information

ELECTRONIC SUPPLEMENTARY MATERIAL

## Data Availability

The datasets generated and/or analyzed during the current study are available in the MIMIC-CXR-JPG repository, https://physionet.org/content/mimic-cxr-jpg/2.1.0/. The data can be obtained after performing the necessary credential process and CITI Data or Specimens Only Research training under the PhysioNet Credentialed Health Data License 1.5.0.

## Abbreviations

AI: Artificial intelligence
BLEU: Bilingual evaluation understudy
LLM: Large language model
LVLM: Large vision language model
METEOR: Metric for evaluation of translation with explicit ordering
ROUGE: Recall-oriented understudy for gisting evaluation
