# Supplementary material for: Enhancing radiology workflows through collaborative AI-assisted chest X-ray reporting using large vision-language models: a proof-of-concept study
Source: Insights Imaging. 2026 Apr 28;17:123. doi: 10.1186/s13244-026-02292-7 (PMC13125563; doi:10.1186/s13244-026-02292-7)
Supplement: Supplementary file 1 — ELECTRONIC SUPPLEMENTARY MATERIAL [file 13244_2026_2292_MOESM1_ESM.pdf]

Enhancing Radiology Workflows through Collaborative AI-Assisted Chest X-Ray Reporting using Large Vision-Language Models: a proof-of-concept study

ELECTRONIC SUPPLEMENTARY MATERIAL

Appendix A: Effect of Visual Input on Completion Quality

To directly test whether RaDialog's suggestions in our collaborative setting depend on the X-ray image rather than only on the report prefix, we conducted an offline ablation experiment. For each study report in our dataset, we segmented the findings section into sentences and constructed short ground-truth prefixes consisting of the first one, two, or three tokens of each sentence. These prefixes simulate intermediate editing states such as "Small opacification…" where a radiologist has already entered the beginning of a sentence but not its full content.

For each prefix, we prompted RaDialog to complete the sentence up to its end under two conditions: (i) RaDialog with visual input, using the standard RaDialog configuration and (ii) LLM-only, where we removed all image embeddings and structured findings and only provided the textual prefix and instruction to continue the findings sentence.

We evaluated the generated completions against the corresponding ground-truth sentences using BLEU-1, METEOR, and ROUGE-L as text similarity metrics, and a CheXbert-based macro F1 score as a measure of clinical label agreement.

Table A1: Report Generation given ground truth prefix token for every report sentence.

**With image input** [95% CI]				
#Prefix Tokens	BLEU-1	METEOR	ROUGE L	CheXbert F1
1	0.50 [0.46, 0.54]	0. 24 [0.22, 0.27]	0.46 [0.42, 0.49]	0.37 [0.29, 0.45]
2	0.55 [0.52, 0.59]	0.29 [0.27, 0.31]	0.54 [0.51, 0.58]	0.47 [0.37, 0.57]
3	0.61 [0.58, 0.65]	0.33 [0.30, 0.35]	0.61 [0.58, 0.65]	0.43 [0.34, 0.53]
**Without image input** [95% CI]				
#Prefix Tokens	BLEU-1	METEOR	ROUGE L	CheXbert F1
1	0.43 [0.39, 0.46]	0.20 [0.18, 0.22]	0.41 [0.37, 0.44]	0.13 [0.07, 0.18]
2	0.51 [0.46, 0.55]	0.25 [0.23, 0.28]	0.52 [0.48, 0.55]	0.22 [0.14, 0.30]
3	0.58 [0.54, 0.62]	0.30 [0.28, 0.33]	0.59 [0.56, 0.63]	0.30 [0.18, 0.40]

As summarized in Table A1, providing the image consistently improves both textual and clinical metrics. For 1-token prefixes, BLEU-1 increases from 0.43 to 0.50 and METEOR from 0.20 to 0.24 when including the image, and CheXbert F1 rises from 0.13 [0.07–0.18] to 0.37 [0.29–0.45]. For 2-token prefixes, CheXbert F1 more than doubles (0.22 [0.14–0.30] vs. 0.47 [0.37–0.57]); even for 3-token prefixes, where the text already carries more context, the image-conditioned model remains clearly superior (0.30 [0.18–0.40] vs. 0.43 [0.34–0.53] in CheXbert F1). These findings demonstrate that the suggestions used in our collaborative tool are clearly conditioned on the image information.

Appendix B: Effect of AI assistance on changes in radiologist interpretation

We performed a manual analysis of non-assisted and AI-assisted reports for the same case and radiologist. First, AI support sometimes helped calibrate severity language more closely to the reference wording (e.g., preserving moderate edema rather than describing as massive edema). Second, AI-assisted reports more often contained explicit, quantitative device positions (e.g., “endotracheal tube tip 4.5 cm above the carina” instead of simply “tracheal tube in situ”), yet could also encourage overspecification of device details, such as the number of pacemaker leads. Third, AI support sometimes shifted the focus of the report: while non-assisted reports sometimes concentrated on a few main findings (e.g., large right-sided pleural effusion), AI-assisted reports more frequently also listed additional, smaller findings (e.g., additional small left-sided effusion) that were consistent with the reference report but not foregrounded by the radiologist. Together, these patterns suggest that AI assistance tends to nudge radiologists toward more explicit and quantitative descriptions and more comprehensive coverage of findings but may also introduce risks of overconfident specificity.

Appendix C: Finding-Based Subgroup Analysis of Writing Time

The effect of AI-assisted writing can vary across complexity and case type. We therefore performed an exploratory stratified analysis by radiographic finding patterns and case complexity using CheXpert-derived labels.

Subgroup definitions: We define pathology findings as the 14 CheXpert labels excluding “No Finding” and “Support Devices” with uncertain labels treated as absent (set to 0), consistent with our main evaluation. We analyze the following sub-groups:

- Normal: No positive pathology findings (excluding “No Finding” and “Support Devices”).
- Low complexity (single finding): Exactly one positive pathology finding.
- High complexity (multi finding): At least k positive pathology findings ($k \in \{3,4\}$).
- Airspace / focal: findings include consolidation, pneumonia or lung opacity.
- Diffuse interstitial findings: findings include Edema.
- Cardiac enlargement: findings include enlarged cardiomediastinum or cardiomegaly.

Methods: For each subgroup, we report mean writing time with and without AI and percent time saved. 95% confidence intervals (CIs) were estimated using a nonparametric bootstrap over studies. Given the small subgroup sizes, results are exploratory and we emphasize effect sizes and CIs rather than binary significance.

Results: As shown in Table C1, the clearest reduction is observed for single-finding studies (≈22.8% mean time reduction; 95% CI 11.7–33.7). Other groups show smaller mean reductions with wider Cis, but all show an average reduction in writing time. The results for different diagnostic subgroups are similar. These subgroup results should be interpreted as descriptive given limited subgroup sizes and future work should explore subgroup effects in studies with larger sample numbers.

Table C1 Writing Time Efficiency Metrics over subgroups

Subgroup	#Report pairs	AI (s) [95% CI]	No AI (s) [95% CI]	Diff. (%)[95% CI] [p]
Normal	26	98.67 [76.36, 121.54]	87.42 [51.62, 125.95]	11.40 [-20.89, 42.04][0.528]
Single finding	39	125.69 [103.44, 147.78]	97.02 [74.30, 122.19]	22.81 [11.73, 33.67][0.002]
>=3 findings	45	126.68 [108.70, 144.56]	114.06 [98.83, 130.35]	9.96 [-5.05, 22.58][0.206]
>=4 findings	23	122.91 [103.83, 142.07]	109.00 [89.82, 131.18]	11.31 [-15.29, 31.73][0.418]
Airspace / focal	51	125.28 [112.96, 137.48]	115.17 [102.95, 127.92]	8.07 [-4.05, 18.59][.205]
Diffuse interstitial	24	113.82 [92.23, 136.30]	104.20 [88.30, 122.50]	8.45 [-22.21, 31.15][0.597]
Cardiac enlargement	57	115.25 [100.08, 131.15]	106.55 [90.05, 123.40]	7.55 [-7.19, 20.86][0.334]
